# Spatial Variation of Survival for Colorectal Cancer in Malaysia

**DOI:** 10.3390/ijerph18031052

**Published:** 2021-01-25

**Authors:** Anis Kausar Ghazali, Thomas Keegan, Benjamin M. Taylor

**Affiliations:** 1 Biostatistics and Research Methodology Unit, School of Medical Sciences, Health Campus, Universiti Sains Malaysia, Kubang Kerian 16150, Kelantan, Malaysia; 2 Lancaster Medical School, Faculty of Health and Medicine, Lancaster University, Lancaster LA1 4YW, UK; t.keegan@lancaster.ac.uk; 3 Blackpool Teaching Hospitals NHS Foundation Trust, Blackpool FY3 8NR, UK; benjamin.taylor.work@gmail.com

**Keywords:** colorectal cancer, survival, spatial survival, spatial modelling

## Abstract

A patient’s survival may depend on several known and unknown factors and it may also vary spatially across a region. Socioeconomic status, accessibility to healthcare and other environmental factors are likely to contribute to survival rates. The aim of the study was to model the spatial variation in survival for colorectal cancer patients in Malaysia, accounting for individual and socioeconomic risk factors. We conducted a retrospective study of 4412 colorectal cancer (ICD-10, C18-C20) patients diagnosed from 2008 to 2013 to model survival in CRC patients. We used the data recorded in the database of the Malaysian National Cancer Patient Registry-Colorectal Cancer (NCPR-CRC). Spatial location was assigned based on the patients’ central district location, which involves 144 administrative districts of Malaysia. We fitted a parametric proportional hazards model in which the spatially correlated frailties were modelled by a log-Gaussian stochastic process to analyse the spatially referenced survival data, which is also known as a spatial survival model. After controlling for individual and area level characteristics, our findings indicate wide spatial variation in colorectal cancer survival across Malaysia. Better healthcare provision and higher socioeconomic index in the districts where patients live decreased the risk of death from colorectal cancer, but these associations were not statistically significant. Reliable measurement of environmental factors is needed to provide good insight into the effects of potential risk factors for the disease. For example, a better metric is needed to measure socioeconomic status and accessibility to healthcare in the country. The findings provide new information that might be of use to the Ministry of Health in identifying populations with an increased risk of poor survival, and for planning and providing cancer control services.

## 1. Introduction

Cancer is a major health burden across the world, with over 14.1 million new cancer cases worldwide in 2012. Of these, around 1.35 million cases (9.6%) are new cases of colorectal cancer. The number of colorectal cancer cases is expected to increase by 80% by 2035, climbing to approximately 2.4 million new colorectal cancer cases and contributing to 1.3 million deaths worldwide [1]. A patient’s survival may depend on several known and unknown factors and it may also vary spatially across a region [2]. Socioeconomic status, accessibility to healthcare and other environmental factors are likely to contribute to survival rates [3,4].

A number of studies have been carried out to investigate and model the spatial variation in survival for various types of cancer. For example, the spatial model of spatial variation in leukaemia survival in north west England [5], the spatial variation in prostate cancer survival in England [6] and an evaluation of the factors affecting the spatial variation in breast cancer survival in Queensland, Australia [7]. Each of these studies suggest that spatial variation in cancer survival exists.

Spatial variation in the survival of colorectal cancer patients has been observed in several studies conducted in developed countries. A study observed disparities in survival across New Jersey among more than 25,000 people diagnosed with colorectal cancer between 1996 and 2003. The lowest survival rates were found mostly in economically deprived areas while those in affluent areas had longer survival times; lack of healthcare accessibility is assumed to be one of the key predictive factors here [8].

Existing research has quantified the geographical variation in survival using a discrete-time multilevel logistic survival analysis for colorectal cancer patients [9]. This research, which included over 400 Statistical Local Area (SLA) regions in Queensland, Australia, found that patients had substantially lower survival rates in rural and deprived areas than patients in urban and affluent areas, after controlling for individual characteristics and cancer stage.

In Spain [10], space-time trends of colorectal cancer mortality risk during the period from 1975 to 2008 were mapped by sex and by two age groups: the middle-aged (50 to 69 years) and the elderly (≥70 years). Their findings demonstrated spatial variation in mortality risk across the region by both sex and age group.

Material deprivation and geographical accessibility to healthcare were found to influence survival in colorectal cancer in a study involving cases from three cancer registries in France and one cancer registry in England [11]. This study showed that both of the above factors were relevant to patient survival, but that the effect differed between the two countries. Material deprivation was significantly associated with cancer survival in England while lack of accessibility to healthcare lead to poorer survival in France. In Korea, a study found that low survival was associated with the late stage at diagnosis found in poorer socioeconomic group patients [12]. Despite the national screening program, those with the lowest socioeconomic status were significantly more often diagnosed with late stage cancer compared with the highest socioeconomic group (the ORs were 1.29; 95% CI; 1.03, 1.61) [12]. On the other hand, the inequalities of survival may also be explained by the fact that the lower socioeconomic status groups received less treatment [13]. The group with the lowest socioeconomic status had a 24% higher risk of death than that of the highest socioeconomic group(hazard ratio 1.24; 95% CI 1.16, 1.32) [13].The findings from these studies suggest that it is important to investigate spatial variation in cancer survival in Malaysia.

There have been several previous studies that have examined the spatial distribution of colorectal cancer incidence in areas of Malaysia, and which have described variation in colorectal cancer incidence, but none have investigated the spatial variation in survival from this disease [14,15,16]. To our knowledge, no studies have examined the epidemiology of this cancer using spatial modelling, and in particular, none have extended research to include the whole of Malaysia or the whole Malaysian population.

Our aim is to model the spatial variation in survival for colorectal cancer patients in Malaysia while accounting for individual and socioeconomic risk factors. We also aim to investigate how individual and socioeconomic factors might affect survival from colorectal cancer, adjusting for spatial location.

Identifying the factors that influence the difference in survival rates across the region may help public health authorities to better plan healthcare delivery, and thus eventually reduce disparities in colorectal cancer survival in Malaysia.

## 2. Materials and Methods

### 2.1. Data

There are two national cancer registries in Malaysia: the National Cancer Registry (NCR) and the National Cancer Patient Registry (NCPR) [17]. Both are managed by the Ministry of Health with the NCR being administered by the Disease Control Division and the NCPR by the National Institute of Health [18].

The NCR captures the data on diagnoses from all regions in Malaysia. Diagnoses are reported to a state registry and from there to the National Registry. However, reporting cases to the state registries from hospitals is voluntary and therefore it is not always completed. However, the NCR is not passive; it conducts active case finding and routine checks. Assessment of the completeness of registration in the NCR is difficult because it is not clear how many of the 165 main hospitals in Malaysia are sending records to the registry, or how accurately diagnoses are recorded even when they are sent [19].

The NCPR collects data on registrations of cancer from participating sites. These participating sites include 34 hospitals that diagnose and treat cancer patients in Malaysia. The objectives of the NCPR are to describe the natural history of cancers and to determine the effectiveness of treatments, to monitor safety, and to evaluate access to treatments. The NCPR collects data on four cancers: colorectal cancer, blood cancers, breast cancer and nasopharyngeal cancers. The NCPR records diagnoses and collects clinical data on risk factors, treatments and patient outcomes. This makes the NCPR data useful for research into the effects of treatments and survival from cancers [20]. This study used data from the National Cancer Registry-Colorectal Cancer (NCPR-CRC) [17].

Our data consisted of 4501 patients with histologically verified primary colorectal cancer diagnosed between 2008 and 2013 (ICD-10, C18-C20). After excluding patients without Malaysian citizenship, patients with negative age and negative survival time, there were 4412 subjects’ data available for analysis.

There are many instances in this dataset where information was missing or incomplete, but these are recorded inconsistently with a variety of indicators such as “missing”, “not applicable”, “not available”, “unknown” or “NA”. These do not always match up with the categories defined for each variable in the data. We therefore decided to combine the various missing data as ”unknown” or ”not applicable” if these categories were stated as such in the patients’ form, or otherwise just as ”missing”. We also obtained advice from our data provider about the uncertain category recorded in the database to justify our decision regarding data categorization. For example, even though the variable ”tumour differentiation” has categories of ”not applicable”, ”not available” and ”missing”, most of the data in these categories did not tally with the data definition. Our data provider clarified this, and assured us that all data in those categories of this variable were actually just ”missing”.

We collected exposure data on ethnicity as Malay and non-Malay. All others were coded as other because of the small numbers. Smoking status was categorised as: non-smoker, former smoker, active smoker and missing status. Education level was classified as “Nil”, “Primary”, “Secondary”, “Tertiary” or “missing”.

Clinical data was recorded as three categories: yes, no and missing. However, cancer stage was recategorised to “Stage I”, “Stage II’, “Stage III”, “Not staged”, and missing. Since the “site of distant metastases” contains many categories, each of which only contained a small number of data points, we decided to combine all the metastases regardless of their specific site. Irrespective of where and when it has been detected, the “presence of distant metastases” was therefore reclassified into yes, no and missing. ”Tumour differentiation” was recorded as “well”, “moderate”, “poor” and “missing”.

The treatment modalities were categorised into four types of treatment received by the patients. They are patients who underwent surgery alone, patients who underwent surgery followed by chemotherapy and/or radiotherapy, patients who underwent chemotherapy and/or radiotherapy and patients who got other alternative treatments or palliative care. Patients without information of the treatment received were recorded as an unknown group.

The specific cause of death provided in the data was not verified and therefore could not be deemed reliable, so, we decided to perform the analysis on all-cause mortality. The data records whether each patient is dead or alive at the end of the study period; we relabelled each patient’s status as either dead or censored. The censored group are patients who were alive until the end of the study period as recorded in the database. 

We obtained ethical clearance from the Ministry of Health Medical Research Ethical Committee (MREC), Malaysia and the Faculty of Health and Medicine Research Ethics Committee (FHMREC), Lancaster University (Ref no: NMRR-15-311-24656(IIR).

### 2.2. Statistical Modeling

The spatial data involved at the district level analysis includes all districts in Peninsular and East Malaysia and was based on polygonal data. The analysis for Peninsular Malaysia and East Malaysia was done separately as they are physically separated. We therefore separated the shapefile of Malaysia into Peninsular Malaysia and East Malaysia.

In order to conduct analysis at the district level, each participant was assigned to their correct district based on the town variable recorded in the data. Each of the 520 unique names of towns seen in the data belong to one of the 144 administrative districts in Malaysia, of which 87 districts are in Peninsular Malaysia and 57 districts are in East Malaysia.

We included a measure of socioeconomic status [21] and the density of hospitals as covariates in our model. The latter was assessed as a proxy for access to healthcare facilities, the lack of which may delay patients seeking or obtaining medical attention early on and which may decrease survival. The socioeconomic status categorisation was based on information from a census in 2000 as a measure of socioeconomic status in Peninsular Malaysia [21]. The index has a positive or negative value. A more positive index for a particular area indicates that the facilities in that area go beyond basic needs, and vice versa for more negative indices [21]. This index was available for 82 of the 87 districts in Peninsular Malaysia, and we used the “autoKrige” function from the ”gstat’ R package to impute the value of the index for the remaining five districts [22]. The autoKrige function implements the technique of ordinary kriging, a method for smoothing spatial data and predicting values for new locations (in this case, the centroids of the districts with missing socioeconomic index).

On the other hand, we created a proxy-measure for accessibility of healthcare using the estimated number of hospitals per unit area (the density), calculated by the “density.ppp” function from the “spatstat” package in R software. Then, we included this density as one of the parameters in the spatial survival model. To model the correlation in space, we used an exponential correlation function, using the distance between the centroids of regions to determine the correlation.

We used the spatial survival model to analyse our spatially referenced survival data. We created our spatial survival model using the “spatsurv” package [23]. The spatsurv package uses parametric models for the baseline hazard function and correlated log-Gaussian frailties to model spatial dependence. The hazard function takes the following form:

*h*(*ti*; *ψ*, *Yi*) *= ho*(*ti; ω*) *exp*{*Xiβ + Yi*},

where *ho* is the baseline hazard function, t is the observed time for the *i* th individual, *Xi* is a vector of covariate values for the *i* th individual, *ψ = h*(*β*,*ω*,*η*) are covariate effects, the parameter of the baseline hazard and the parameter of the covariance function of a spatially latent Gaussian field *Y*, respectively. *Yi* is the value of the field at the location of individual *i.*

In the Bayesian model, a prior density for the parameter of interest was inputted and the data modified the prior by using the likelihood to arrive at the posterior.The spatsurv package uses a Markov chain Monte Carlo (MCMC) algorithm to perform Bayesian inference for the parametric proportional hazards model. The idea is to use MCMC to draw samples from the posterior and estimate the parameters in our model. The spatsurv package implements the Markov chain Monte Carlo (MCMC) inferential algorithms because although they are typically slower than approximate methods, such as those based on the Laplace approximation for example, they deliver an unbiased, joint inference for all model parameters and are relatively easily extensible to wider model classes with additional hierarchies [23]. We looked for evidence of satisfactory convergence and mixing in the MCMC chain by considering the mcmcplot of *β*, *ω*, *η* and *Y*. We compared plots of prior and posterior to check that our data were sufficient to allow identifiability of the parameters in our model. The model has been fitted using three different distributions for the baseline hazards: Weibull, Exponential and B-spline. The models were compared using the Watanabe-Akaike information criterion (waic) value.

We plotted the posterior baseline and cumulative hazard for each model, as well as the spatial covariance function and correlation against distance, for Peninsular Malaysia and East Malaysia. The plots of the posterior spatial correlation function show how similar the hazard is across space, and how fast that similarity decays. A correlation plot with a fast drop (small Ø) shows that there is little dependence between the hazard and distance. On the other hand, if the correlation plot has a slow drop, this shows that there is strong spatial dependence in the hazard or the risk of death from colorectal cancer is highly associated with place. It means that even though there may be a large distance between places, the correlation in their hazard is high. The interpretation of Ø is that for distances over Ø apart, there is little dependence on space.

We also mapped the probability that the covariate-adjusted relative risk exceeds certain thresholds. These plots represent the risk over space that is not accounted for by the covariates in our model. All analysis were carried out using R software.

## 3. Results

We produced two spatial survival models, one for Peninsular (West) Malaysia and one for East Malaysia. Table 1 shows the parameter estimates for the hazard of death from the covariates used in these models. The covariates with a credible interval of hazard ratio(HR) that are marked with an asterisk (*) are significantly associated with the hazard of death from colorectal cancer in our spatial survival model. 

After controlling for spatial location and socioeconomic factors, cancer staging still plays an important role in determining the risk of death from colorectal cancer in Malaysia. Patients diagnosed at Stage IV had six times (median 6.37, 95% CRI (4.34, 9.75)) and seven times (median 7.33, 95% CRI (2.99, 24.00)) higher risk of death from colorectal cancer in West and East Malaysia, respectively, than patients diagnosed at Stage I. Each year of increase in age led to a slight increase (median 1.01, 95% CRI (1.01, 1.02)) in the relative risk of death. Other factors that significantly affect survival in colorectal cancer patients in Malaysia were ethnicity, tumour differentiation and the presence of distant metastases. We included two parameters to represent the socioeconomic distinctions in the population; the density of the hospitals in each district and the middle-class household item index (socioeconomic index). However, the socioeconomic index was only available for Peninsular Malaysia. Both of these variables showed an association with a decrease in the risk of death as the value of the parameters increases, however neither result was significant.

The table shows the hazard of death from colorectal cancer for the variables chosen in the spatial survival model. It is represented by the median HR and 95% credible interval.

**Figure 1:**
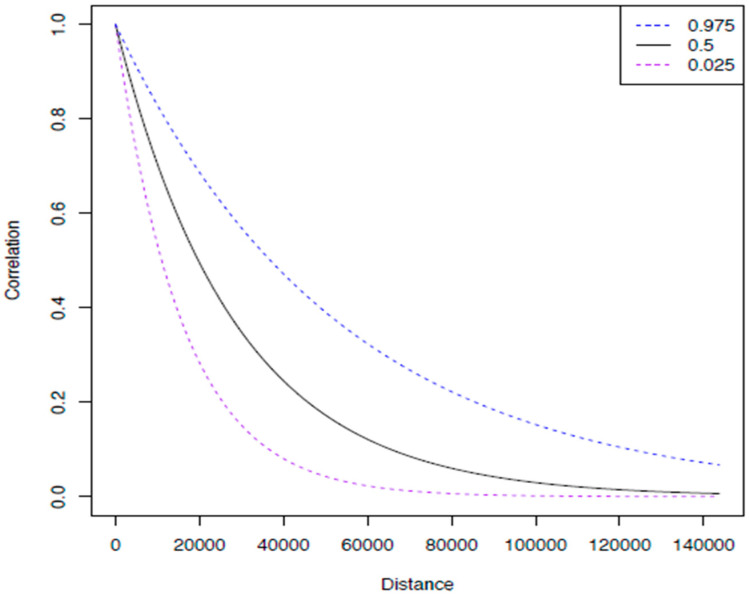
The figure above shows the plot of the posterior spatial correlation function for Peninsular Malaysia. It shows that cases within a distance of less than 17 km (Ø) had a high correlation of hazard in space. The correlation of hazard starts to decrease when the distance for cases is more than 17 km apart; this is supported by the Ø value shown in Table 1.

**Figure 2:**
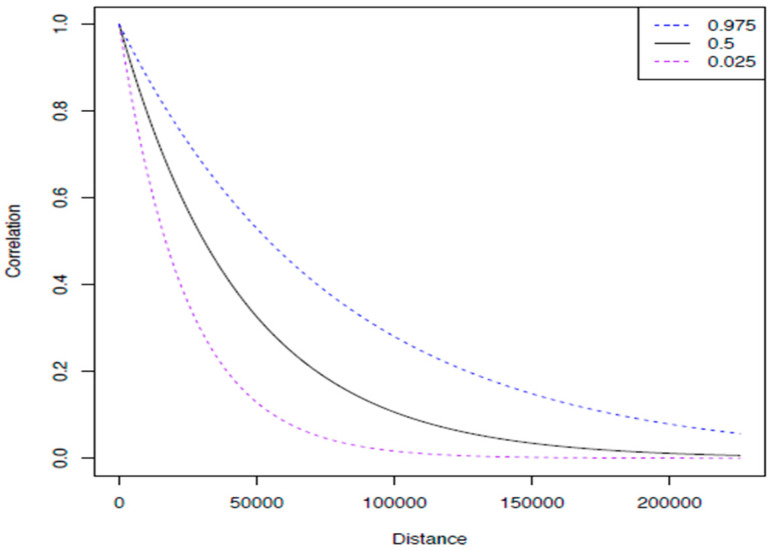
The figure above shows the plot of the posterior spatial correlation function for East Malaysia. It shows that the cases within a distance of less than 45 km (Ø) had a high correlation of hazard in space. The correlation of hazard starts to decrease when the distance of cases was more than 45 km apart; this is supported by the Ø value shown in Table 1.

The spatial correlation function plot (Figure 1 and Figure 2) shows how similar the hazard is across space, and how fast that similarity decays.

**Figure 3:**
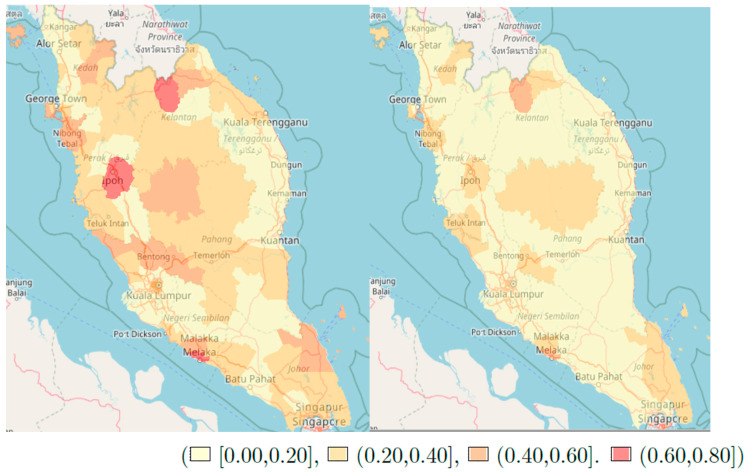
The risk map for the probability of exceedance risk of hazard of 1.1 and 1.25 in Peninsular Malaysia: ℙ[exp(Y) > 1.1] (left) and ℙ[exp(Y) > 1.25] (right).

Figure 3 shows the risk map (leaftlet plot) for the probability of exceedance risk of hazard of 1.1 and 1.25 in Peninsular Malaysia. These plots show P[exp(Y) > 1.1] and P[exp(Y) > 1.25]. Three regions in Peninsular Malaysia were identified as having a higher probability that the hazard of death would exceed 1.1, that is, those with a probability greater than 0.75 of exceeding the stated hazard. The probability starts to lessen when we increase the exceedance threshold to 1.25.

**Figure 4:**
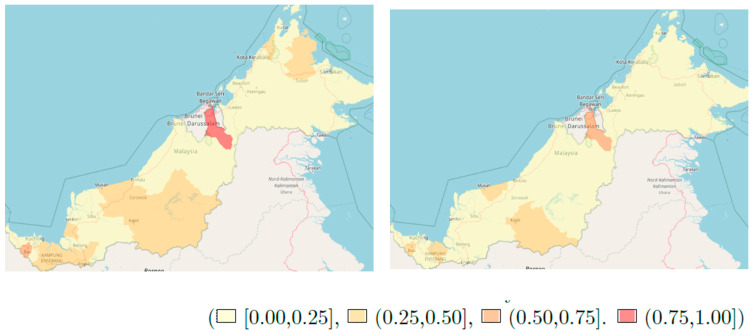
The risk map for the probability of exceedance risk of hazard of 1.25 and 1.5 in East Malaysia: P[exp(Y) > 1.1] and P[exp(Y) > 1.25].

Figure 4 shows the risk map for the probability of exceedance risk of hazard of 1.25 and 1.5 in East Malaysia. East Malaysia has two states, Sabah and Sarawak. The areas with the highest probability of exceeding the stated hazard of death in East Malaysia are all located in Sarawak. There was one district in Sarawak, Limbang, that was highly likely to have a hazard of death exceeding 1.25. Upon increasing the threshold to 1.5, only one part of the Sarawak region still had a probability of exceedance of between 0.50 to 0.75, and none had a probability greater than 0.75.

## 4. Discussion

In this study, we investigated the survival model of colorectal cancer in Malaysia, and incorporated geographical location. Our findings show that there is spatial variation in survival prognoses, or the hazard of death, for colorectal cancer in Malaysia even after adjusting for individual-level and area-level covariates. Cancer staging, tumour differentiation and the presence of distant metastases have a significant effect on survival for colorectal cancer patients. However, we also found that Malays had a significant 26% higher risk of dying from colorectal cancer than non-Malays. An increase in age slightly increased the risk of death from colorectal cancer.

We found that a high socioeconomic index in an area did not significantly affect the risk of death from colorectal cancer. Our findings were similar to those of [24] who found that the socioeconomic status of the population did not significantly influence outcomes in patients with colorectal cancer. In comparison, a systematic review by [25] found that socioeconomic status had a significant impact on survival of colorectal cancer, where the risk of death was greater among patients with low socioeconomic status. Regarding socioeconomic status, recall that our measure, based on the 2000 census, was aggregated to district level. Hence a potential explanation for not observing a significant effect may be due to the presence of ecological bias, that is, our measure of socioeconomic status did not pertain to individuals. In fact, considering that education is often directly related to socioeconomic status, our results do show evidence that higher socioeconomic status is protective. However, further research in this area is required, and a more finely-resolved spatial map of deprivation could help us to better identify this effect.

It is possible that there is a positive association between our variable “education”, an individual-level variable, and our socioeconomic index, which is an area-level factor measure with education as one of its domains. We think that these two measures are not likely to be well correlated for the following reasons. The socioeconomic index includes a measure, at an area level, of the proportion of the population with tertiary education in a district (area level). Education is one of five domains by which the socioeconomic measure is comprised. We realize that education might drive socioeconomic status, but it is not the sole driver of socioeconomic status. The widely used UK Index of Multiple Deprivation (IMD) is comprised of seven domains, one of which is based on education, and the index measures small areas across England, called Lower-Layer Super Output Areas (LSOAs) [26].

The education variable in the model represents the individual education level of the patients in this study, which is classified into five categories, nil, primary, secondary, tertiary and missing status. Thus, we think that the education and the SE index variable in the model represent different things. To see if this is likely, we checked if there was any correlation between the two variables but it was not significant with a correlation coefficient of ρ = 0.01.

We noticed that there were patients that had changed their address after diagnosis, but we decided not to take the distance of the patients’ addresses to the hospital as our measure of accessibility to healthcare. Address at diagnosis is important as an “exposure” or proxy for unmeasured exposures, but we had no record of length of residence at the address at diagnosis, which leaves open the likelihood of misclassifying cases by exposure to place. For example, people move house or job for many reasons, but sometimes for health reasons. They may, for example, move nearer a hospital when ill, or away from an exposure when concerned and there is evidence that population movement can lead to misclassification in epidemiological studies, such as when a birth address is used in studies of birth defects [27]. Instead, we decided to look at how the density of the hospitals in the area affected the patients’ survival in our study.

The relationship between survival and distance to the treatment hospital is not clear cut: it is not necessarily the closest hospital to patients that they will choose to go to seek treatment and for this reason, we used the smoothed density(number of hospitals per unit area) as a covariate in our model. We expected the coefficient of this covariate to be positive, since, in places where there is a greater concentration of hospitals, patients tend to get diagnosed quicker as early symptoms are recognised and acted upon. We found that greater accessibility to healthcare decreased the risk of death from colorectal cancer but the effect was not significant in our study, which contradicts a previous study reporting that lack of access to healthcare was significantly associated with being diagnosed at a more advanced stage of colorectal cancer [28], which we know adversely affects patient survival. Different ways of assessing accessibility to healthcare may influence the direction and significance of the effect. For example, the previously mentioned study measured the shortest time taken to travel to the nearest appropriate health facility, while our study measured the number of hospitals per unit area. Furthermore, other factors that could be mediators to the accessibility to healthcare, such as the transportation system, affordability of care and cultural barriers were not considered in our study.

Three areas were found to have a high risk of death (where the risk of death > 1.1 relative to other areas in Peninsular Malaysia). They were Jeli district in the North-East, Kinta district in the North-West and Melaka Tengah located in the West of Peninsular Malaysia. In East Malaysia, Sabah had better survival compared to Sarawak as shown by the probability of exceedance risk maps. The reasons for these differences could be such things as delay in chemotherapy treatment [29] and comorbidities [30], which were not assessed in our study, and this would be an interesting direction for further work in this area.

We have a limitation as Malaysia does not have a formal socioeconomic status instrument that is consistent across the population (for example, the UK have the Index of Multiple Deprivation, IMD) [26]. Currently, the Malaysian Statistics Department only produces data on income at the district level; data on income at finer levels does not exist. We hope that in future research, it will be possible to consider household income and employment status as additional indications of socioeconomic status.

The spatial analysis method used here has several strengths. First it allows us to combine data from the individual level with data from aggregated levels and map our findings. It also allows us to estimate and map the effect of unobserved environmental confounders through the use of a spatially correlated random effect terms. We applied MCMC for our analysis as it delivers full joint inference for all model parameters. Some limitations to be considered is that this analysis assumes a particular model form and correlation structure for the spatial variation and that it can be difficult in practice to identify the parameters of the spatial process. Attaining good convergence and mixing of MCMC can be difficult without access to bespoke software but again this is not an issue for us. Despite these limitations, this is the first study to examine the variation in survival for colorectal cancer in Malaysia. Having controlled for the potential individual and area-level factors, we found there is variation in the survival or risk of death from colorectal cancer in the population.

## 5. Conclusions

Our findings indicate there is wide spatial variation in colorectal cancer survival across Malaysia, after controlling for individual and area-level characteristics. Better healthcare provision and higher socioeconomic index in the districts where patients live decrease the risk of death from colorectal cancer, although the associations were not statistically significant in this study. To obtain reliable SES data from individuals would require a range of questions to be answered as part of the initial data entry form. This might seem an unnatural thing to try to elicit routinely as part of a colorectal cancer diagnosis by medical practitioners. Similar comments apply to our chosen measure of healthcare accessibility; again, this information would be better obtained from each patient.

In the future, the following suggestions may improve the quality of data and its resulting analysis. Reliable measurements of environmental factors are needed to provide good insight into the effects of potential risk factors for the disease. For example, a better metric is needed to measure the socioeconomic status and accessibility to healthcare in the country. Ensuring complete, accurate and consistently recorded data in the National Cancer Registry database is vital for reliable analysis and meaningful results. Perhaps ascertainment of active cases across the country and also better training of staff that interact with the database is required in order to minimise the amount of missing data.

This study will provide new input for the Ministry of Health to target the population with a high risk of poor survival in providing cancer control services and to enhance health activities that are cancer-related in order to improve survival in the population.

## Figures and Tables

**Table 1:** Table of parameter estimates from the fitted spatial survival model.

Covariates	n	Peninsular Malaysia Median HR (95% CRI) **	East Malaysia Median HR (95% CRI) **
**Age**	4412	1.01 (1.01, 1.02) *	1.01 (1.01, 1.02) *
**Sex**			
Female	1894	Reference	
Male	2470	1.05 (0.93, 1.19)	0.96 (0.76, 1.24)
Missing	48	0.81 (0.40, 1.42)	0.60 (0.02, 5.29)
**Ethnicity**			
Non Malay	2489	Reference	
Malay	1901	1.26 (1.13, 1.43) *	1.47 (1.05, 2.01) *
Missing	22	1.24 (0.20, 4.58)	1.53 (0.27, 5.87)
**Education**			
Nil	399	Reference	
Primary	553	0.92 (0.72, 1.17)	1.00 (0.68, 1.46)
Secondary	651	1.00 (0.78, 1.27)	1.01 (0.68, 1.47)
Tertiary	195	0.63 (0.43, 0.88) *	0.60 (0.32, 1.10)
Missing	2614	1.03 (0.85, 1.28)	0.82 (0.61, 1.18)
**Smoking**			
Non Smoker	1543	Reference	
Former Smoker	528	1.17 ( 0.97, 1.40)	1.45 (0.99, 2.00)
Active smoker	423	1.13 ( 0.93, 1.37)	1.12 (0.76, 1.63)
Missing	1918	1.03 (0.90, 1.19)	1.09 (0.80, 1.50)
**Staging**			
Stage I	215	Reference	
Stage II	600	1.65 (1.09, 2.57) *	2.04 (0.81, 6.85)
Stage II	802	3.17 (2.13, 4.83) *	3.63 (1.43, 10.90) *
Stage IV	647	6.37 (4.00, 9.75) *	7.33 (2.99, 24.00) *
Not staged	620	2.79 (1.88, 4.46) *	5.06 (2.03, 16.00) *
Missing	1528	4.14 (2.82, 6.45) *	3.91 (1.56, 12.70) *
**ICDSite**			
Colon	2394	Reference	
Rectum	631	1.05 (0.90, 1.23)	1.32 (0.95, 1.84)
Rectosigmoid	1379	1.06 (0.94, 1.19)	1.16 (0.89, 1.49)
Missing	8	0.29 (0.01, 1.52)	
**TumourDiff**			
Well	358	Reference	
Moderate	2497	1.17 ( 0.94, 1.46)	1.77 (1.08, 3.01) *
Poor	161	2.45 (1.81, 3.40) *	2.99 (1.46, 5.88) *
Missing	1396	1.59 (1.28, 1.99) *	2.84 (1.73, 4.97) *
**Metastasis**			
No	1922	Reference	
Yes	1521	1.40 (1.21, 1.62)	1.59 (1.19, 2.13)
Missing	969	1.47 (1.21, 1.77)	1.39 (0.93, 2.11)
**Treatment**			
Surgery Alone	1658	Reference	
Surg, Chemo, radio	1454	0.87 (0.76, 0.99)	0.98 (0.75, 1.27)
Radio, Chemo	437	1.11 (0.91, 1.35)	1.22 (0.83, 1.81)
Other	140	1.84 (1.41, 2.38)	2.36 (0.99, 4.96)
Unknown	723	1.13 (0.95, 1.33)	1.56 (0.99, 2.35)
**Diabetes**			
No	3021	Reference	
Yes	982	1.12 (0.99, 1.28)	1.08 (0.77, 1.43)
Missing	409	0.88 (0.72, 1.06)	0.73 (0.28, 1.56)
**Hosp. density**		0.97 (0.81, 1.143)	0.69 (0.03, 13.70)
**SE Index**		0.85 (0.53, 1.39)	
**Ø**		16384 (5828, 44570)	44602 (24370, 78650)

* significant estimates; ** CRI: credible interval. ** Median Hazard Ratio.

## Data Availability

Restrictions apply to the avaialability of these data. Data was obtained from the National Cancer Patient Registry Colorectal Cancer(NCPR-CRC) database with permission and ethical approval from Medical Research Ethical Committee (MREC), Malaysia (Ref no: NMRR-15-311-24656(IIR).
